# Obstruction of the formation of granulation tissue leads to delayed wound healing after scald burn injury in mice

**DOI:** 10.1093/burnst/tkab004

**Published:** 2021-04-29

**Authors:** Yunxia Chen, Xiaorong Zhang, Zhihui Liu, Jiacai Yang, Cheng Chen, Jue Wang, Zengjun Yang, Lei He, Pengcheng Xu, Xiaohong Hu, Gaoxing Luo, Weifeng He

**Affiliations:** 1 State Key Laboratory of Trauma, Burn and Combined Injury, Institute of Burn Research, Southwest Hospital, Third Military Medical University (Army Medical University), Chongqing 400038, China; 2 Chongqing Key Laboratory for Disease Proteomics, Chongqing 400038, China; 3 Department of Dermatology, Southwest Hospital, Army Military Medical University, Chongqing, China; 4 Department of Osteopathic Medicine, Southwest Hospital, Army Military Medical University, Chongqing, China

**Keywords:** Burn wound healing, Second-degree deep scald, Third-degree scald, Re-epithelialization, Granulation tissue, Healing quality

## Abstract

**Background:**

Delayed wound healing remains a common but challenging problem in patients with acute or chronic wound following accidental scald burn injury. However, the systematic and detailed evaluation of the scald burn injury, including second-degree deep scald (SDDS) and third-degree scald (TDS), is still unclear. The present study aims to analyze the wound-healing speed, the formation of granulation tissue, and the healing quality after cutaneous damage.

**Methods:**

In order to assess SDDS and TDS, the models of SDDS and TDS were established using a scald instrument in C57BL/6 mice. Furthermore, an excisional wound was administered on the dorsal surface in mice (Cut group). The wound-healing rate was first analyzed at days 0, 3, 5, 7, 15 and 27, with the Cut group as a control. Then, on the full-thickness wounds, hematoxylin and eosin (H&E) staining, Masson staining, Sirius red staining, Victoria blue staining and immunohistochemistry were performed to examine re-epithelialization, the formation of granulation tissue, vascularization, inflammatory infiltration and the healing quality at different time points in the Cut, SDDS and TDS groups.

**Results:**

The presented data revealed that the wound-healing rate was higher in the Cut group, when compared with the SDDS and TDS groups. H&E staining showed that re-epithelialization, formation of granulation tissue and inflammatory infiltration were greater in the Cut group, when compared with the SDDS and TDS groups. Immunohistochemistry revealed that the number of CD31, vascular endothelial growth factor A, transforming growth factor-β and α-smooth muscle actin reached preferential peak in the Cut group, when compared with other groups. In addition, Masson staining, Sirius red staining, Victoria blue staining, Gordon-Sweets staining and stress analysis indicated that the ratio of collagen I to III, reticular fibers, failure stress, Young’s modulus and failure length in the SDDS group were similar to those in the normal group, suggesting that healing quality was better in the SDDS group, when compared with the Cut and TDS groups.

**Conclusion:**

Overall, the investigators first administered a comprehensive analysis in the Cut, SDDS and TDS groups through *in vivo* experiments, which further proved that the obstacle of the formation of granulation tissue leads to delayed wound healing after scald burn injury in mice.

Highlights

- The wound-healing rate, re-epithelialization, formation of granulation tissue and inflammatory infiltration were greater in the Cut group, when compared with the SDDS and TDS groups.
- The number of blood vessels in the Cut group reached preferentially peaking, when compared with the SDDS and TDS groups.
- The ratio of collagen I to III, reticular fibers, failure stress, Young’s modulus and failure length in the SDDS group were closer to the normal group, suggesting that the healing quality of the SDDS group was better, when compared with the Cut and TDS groups.

## Background

Delayed wound healing remains a common but challenging problem after skin injury, such as burn injury, trauma, scald, diabetes, etc. Scald burn injury, including but not limited to second-degree deep scald (SDDS) and third-degree scald (TDS), leads to pathological scars and a deformed appearance; and serious scald burn injury may cause skin dysfunction, such as hair loss^[^^1^^,^^2^^]^. In particular, the cutaneous damage with a large area can lead to the impairment of wound healing and poor prognosis, and even result in death^[^^3^^]^. However, to date, the detailed assessment and mechanism of delayed wound healing following scald burn injury remains unknown in mammals. Hence, a systematic evaluation may be required for SDDS and TDS.

It has commonly been considered that wound healing following cutaneous injury, which is a complex and dynamic process, primarily comprises four successive, but overlapping phases: hemostasis, inflammation, proliferation, and remodeling^[^^4^^]^. In general, skin healing requires the well-orchestrated integration of complex biological and molecular events, such as cell proliferation and migration, angiogenesis, collagen synthesis and deposition, wound contraction, and remodeling^[^^5^^]^. In the early phase after the damage to skin, the exposed sub-endothelium tissue and collagen activates platelet aggregation, which further results in degranulation, and releases growth factors and chemokines to form a blood clot^[^^6^^]^. Next, a number of inflammatory cells, including neutrophils and macrophages, will appear at the injury site, in which the debris, bacteria and damage tissues are gradually cleansed and phagocytized^[^^7^^]^. In the subsequent proliferation phase, fibroblasts, angiogenesis and re-epithelialization take place in the wound. Also, fibroplasia and angiogenesis occur concurrently in a compactly orchestrated manner, in order to form the extracellular matrix and granulation tissue^[^^8^^]^. Although fibroblasts play a key role in wound healing by secreting collagen and fibronectin to create temporary scaffolds promoting wound repair, the aberrant proliferation of fibroblasts and the excessive synthesis of collagen leads to the formation of hypertrophic scars, which greatly compromise the healing quality^[^^5^^,^^9^^]^. In addition, collagen type I and type III are excessively synthesized in the scar area during the process of wound repair, leaving a poorly organized dense collagen-rich matrix that is often highly contracted by myofibroblasts^[^^10^^]^. Therefore, the present study evaluates the speed and quality of wound healing via the formation of granulation tissue, angiogenesis, collagen synthesis, inflammation infiltration, and the mechanical property of skin, following cutaneous damage.

In order to investigate the mechanism of delayed wound healing following burn scald injury, the present study initially established the Cut, SDDS and TDS models in C57BL/6 mice, and compared the rate and quality of wound healing among these three groups. Furthermore, various indicators, including re-epithelialization, angiogenesis, granulation tissue and collagen, during wound healing were analyzed, which may provide some clinical value for the treatment and prognosis of severe burn patients in the future.

## Methods

### Animals

A total of 100 C57BL/6 mice (male, 6–8 weeks old; body weight, 20–25 g) were purchased from the Experimental Animal Department of Army Medical University (Third Military Medical University) with animal license: SCXK (J) 2007–017. All animal procedures were approved by the Animal Ethics Committee of Army Military Medical University.

### Preparation of full-thickness excisional wound

The hair on the dorsal surface of the mice was removed two days before the experiment. Then, a total of 24 C57BL/6 mice were placed under anesthesia using 1% pentobarbital (Sigma, USA) via intraperitoneal injection (0.5–1.0 mL/100 g of body weight). After disinfecting with 75% ethyl alcohol, an excisional wound (1 cm in diameter) was administered (full-thickness wounds) on the dorsal surface (Cut group). Then, the wound area was recorded daily through macroscopic digital photographs, and these were calculated at each time point as a percent relative to day zero, until complete closure. All wounds were left uncovered, and mice were individually housed with sterile bedding.

### Preparation of the scald burn model

The scald burn model was established according to previous protocols^[^^11^^]^. Briefly, the shaved mice were anesthetized by intraperitoneal injection of 1% pentobarbital. For the induction of the scald burn injury, the probe (1 cm in diameter) of the scald instrument (Zhongshidichuang Technology Development Co. Ltd, Beijing, China) was pre-heated (85°C) and applied on the dorsal surface of each mouse, and maintained in contact with the skin for five seconds to produce a nonlethal SDDS group. For the TDS group, the probe was pre-heated to 95°C, and maintained in contact with the skin for nine seconds. The wound area was recorded daily by photographs, and these were calculated at each time point as a percent relative to day zero, until complete closure. The number of animals assigned to each group was 38 C57BL/6 mice.

### Wound-healing rate analysis

Briefly, photographs of the wounds were taken at an equal distance at days 0, 3, 5, 7, 15 and 27 after surgery, respectively. The area of wound healing was measured using ImageJ software (National Institutes of Health [NIH], USA), and the wound-healing rate was calculated using the following formula: wound-healing rate (%) = (AW_i_—AW_n_)/AW_i_ × 100%; where: AW_i_ represents the area of the initial wound (the actual size after wound creation) and AW_n_ represents the area of the wound healing at post-operation at different time points.

### Hematoxylin–eosin staining

Mice were sacrificed via intraperitoneal injection excessive 1% pentobarbital. Then, the wound tissues were carefully harvested at days 3, 7, 15 and 27 after surgery, respectively. Immediately, all samples were fixed with 4% formaldehyde, embedded in paraffin and sectioned. Subsequently, the sections were stained with hematoxylin and eosin (H&E). The newly formed epidermis, granulation tissue, angiogenesis and inflammatory cell infiltration around the wound were observed under a microscope.

### Immunohistochemistry

The paraffin sections were deparaffinized, rehydrated, blocked and incubated with the primary antibody at 4°C overnight. Then, the sections were incubated with biotinylated goat-anti-rabbit IgG antibody (Zhongshan Biology Co. Ltd, China) for 15 minutes at room temperature and subsequently incubated with avidin peroxidase reagent (Zhongshan Biology Co. Ltd, China). Afterward, the counterstaining was carried out with hematoxylin (Beyotime, China) and observed using a microscope. The primary antibodies were as follows: anti-CD31 (1:100, Abcam, UK), anti-α-smooth muscle actin (α-SMA) (1:150, Abcam, UK), anti-vascular endothelial growth factor A (VEGFA) (1:100, Abcam, UK), anti-interleukin 17A (IL-17A) (1:500, Abcam, UK), anti-interferon γ (IFN-γ) (1:100, Abcam, UK), anti-transformation growth factor-β (TGF-β) (1:200, Abcam, UK).

### Analysis of collagen, reticular and elastic fibers

In the skin, type I collagen accounts for about 90% of the total collagen content and often forms thicker fibrous bundles, while type III collagen forms a fine proto-fibrous network^[^^12^^]^. These two collagens provide superior properties for maintaining the structure and mechanics of skin^[^^13^^]^. Therefore, to some extent, the ratio of collagen type I to III and the percent of reticular and elastic fibers could be used to assess the healing quality after skin injury. The paraffin sections were deparaffinized, rehydrated and stained by Masson and Sirius red according to the manufacturer’s protocol. Masson staining was administered to dye the collagen fibers, while Sirius red staining was used to stain the collagen type I and III. Furthermore, the reticular fibers were dyed by using a reticular fiber staining kit, while elastic fibers were stained using Victoria blue. All staining kits were purchased from Beijing Solaibao Technology. The aforementioned staining sections were observed using a light microscope. The average optical density (AOD) was calculated and analyzed using ImageJ software (NIH, USA).

### Measurement of the mechanical properties

The mechanical properties of skin following wound repair were measured by the tensile failure test using the Instron 5567 materials testing system (Instron, USA). Briefly, the samples were cut into dumbbell shapes and fixed on the sensor of the universal material testing machine. Then, the upper and lower clamps were adjusted on the same vertical horizontal plane, in order to ensure that the stress direction of the skin was vertical. Finally, the upper and lower clamps were tightened to prevent the sample from sliding out during the experiment. When the test began, the samples were elongated until avulsion with the constant speed 50 mm/min, and the data was analyzed using GraphPad Prism 8.0 software (GraphPad Software LLC, USA).

### Statistical analysis

All data obtained from the three independent experiments were presented as mean ± SD. The statistical differences were calculated by one-way analysis of variance (ANOVA), two-way ANOVA using SPSS 20.0 software (IBM, USA), and the graphs were prepared using GraphPad Prism 8.0 software. A *p* value < 0.05 was considered statistically significant.

## Results

### The wound-healing rate of the Cut group was significantly higher than that of the SDDS and TDS groups

**Figure 1.**
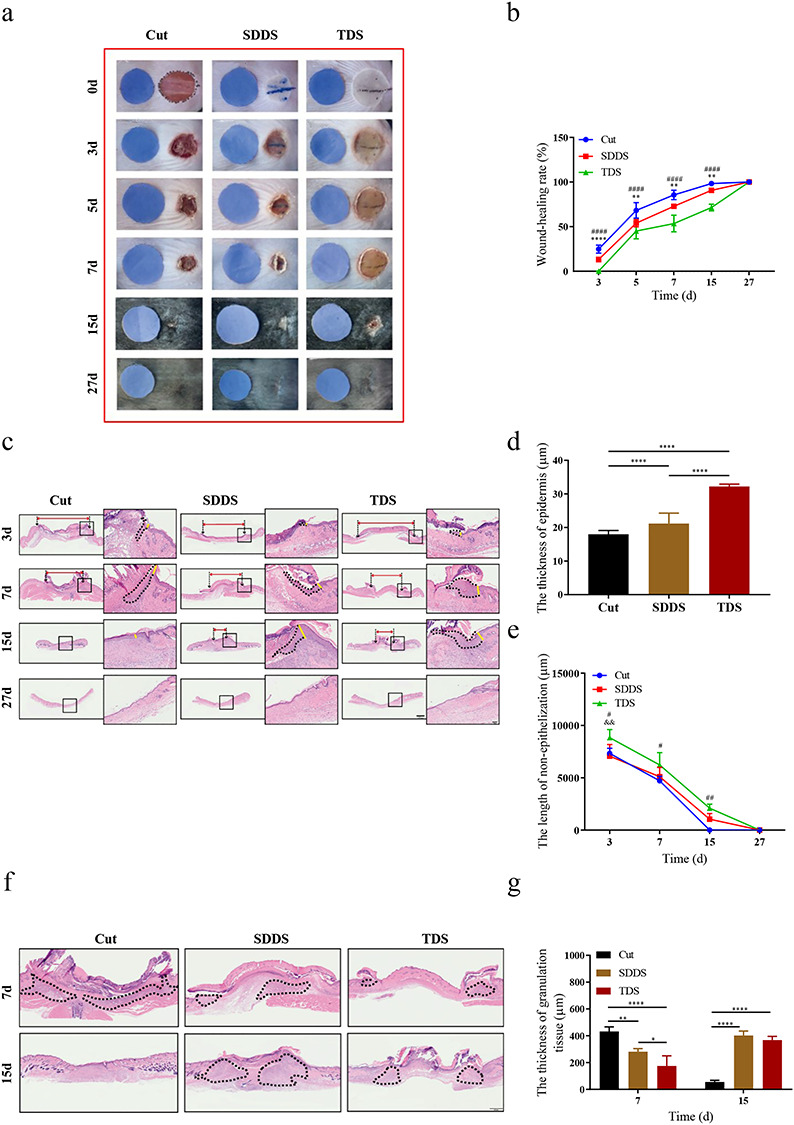
The difference of wound-healing rate in the Cut, SDDS and TDS groups at days 0, 3, 7, 15, and 27 after skin injury **(a)** Representative wound images; **(b)** statistical analysis of the percentage of wound-healing area in the Cut, SDDS and TDS groups at days 3, 7, 15 and 27. *Cut *vs* SDDS, #Cut *vs* TDS. **(c)** H&E staining was used to analyze the differences of re-epithelialization (the red dotted line indicates the unhealed length, the yellow dotted line indicates the epithelial thickness, and the black dotted line represents the epithelium tongue). Scale bar: 200 and 50 μm, magnification times: 10× and 40×. **(d)** Statistical analysis of epithelial thickness and **(e)** unhealed length among three groups at different time points. *Cut *vs* SDDS, #Cut *vs* TDS, &SDDS *vs* TDS. **(f)** H&E staining was used to analyze the thickness of granulation tissue (the black dotted line represented the granulation tissue) in the Cut, SDDS and TDS groups at different time points. Scale bar: 500μm, magnification times: 20×. **(g)** Statistical analysis of the thickness of granulation tissues among three groups at different time points. *Cut *vs* SDDS. All data from three independent experiments were presented as mean ± SD. ^*^p < 0.05, ^**^p < 0.01, ^***^p < 0.001, ^****^p < 0.0001; ^#^*p* < 0.05, ^##^*p* < 0.01, ^####^*p* < 0.0001; ^&&^*p* < 0.01. *SDDS* second-degree deep scald, *TDS* third-degree scald, *H&E* hematoxylin and eosin

In order to evaluate the healing speed of skin wounds, the investigators analyzed the healing rate at different time points (days 0, 3, 5, 7, 15 and 27) after injury (Figure 1a). The results revealed that the wound-healing rate of the Cut group was significantly higher than that of the SDDS and TDS groups (Figure 1b), suggesting that the healing speed was faster, when compared with the other two groups. For the harvested specimens on the 3rd, 7th, 15th and 27th days, H&E staining was performed and used to assess the re-epithelialization, including the epithelial thickness and granulation tissue, among the three groups (Figure 1c). The present results revealed that the epithelial thickness in the TDS group at day 15 was significantly thicker, when compared with the Cut and SDDS groups (Figure 1d). This further proved the re-epithelialization in the Cut group, which was significantly accelerated, when compared with the SDDS and TDS groups (Figure 1e). This implies that re-epithelization in the SDDS and TDS groups was impaired due to the scald burn damage. Furthermore, granulation tissues were observed through H&E staining on days 7 and 15 (Figure 1f). These present results revealed that the granulation tissue was markedly thicker and reached a maximum at day 7 following surgery, when compared with the SDDS and TDS groups, while it reached a peak at day 15 in the SDDS and TDS groups, when compared with the Cut group (Figure 1g). These results indicate that the obstacle to wound healing in the SDDS and TDS groups may have resulted from the impairment of granulation tissue formation.

### Delayed angiogenesis in the SDDS and TDS groups when compared with the Cut group

**Figure 2.**
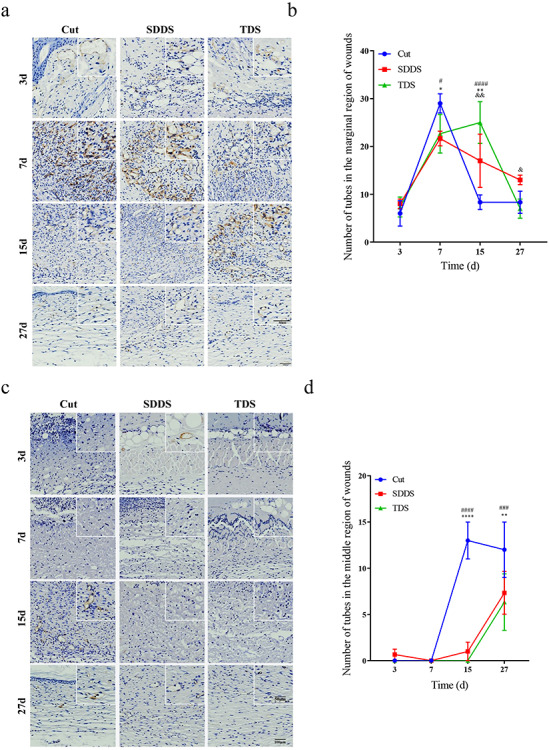

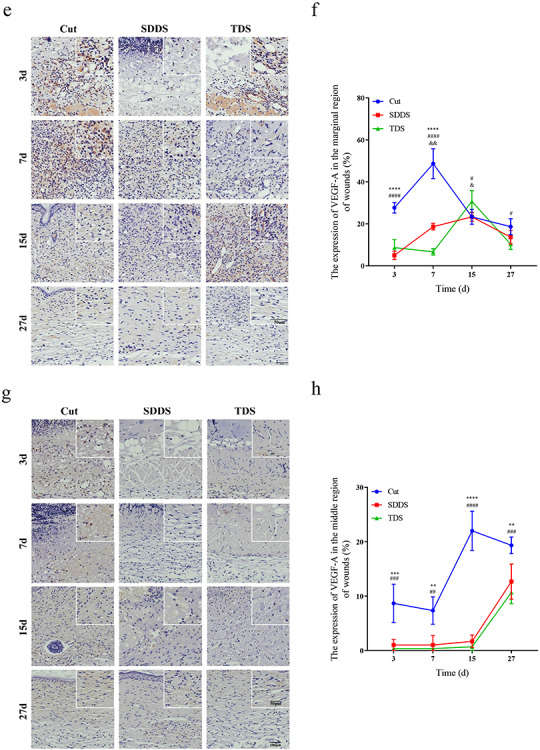
The variation of neovascularization in the Cut, SDDS and TDS groups on the 3rd, 7th, 15th and 27th days after surgery. **(a)** The distribution of blood vessels (CD31 positive area) by immunohistochemistry staining in the marginal region of wounds at days 3, 7, 15 and 27. Scale bar: 100μm and 50μm, magnification times: 40× and 100×. **(b)** Statistical analysis of the amount of neovascularization in the marginal region of wounds at different time points. *Cut *vs* SDDS, #Cut *vs* TDS, &SDDS *vs* TDS. **(c)** Immunohistochemistry staining showed that the distribution of blood vessels using at the middle region of wounds at days 3, 7, 15 and 27. **(d)** Statistical analysis of the amount of neovascularization at the middle region of wounds in the Cut, SDDS and TDS groups at different time points. *Cut *vs* SDDS, #Cut *vs* TDS, &SDDS *vs* TDS. **(e)** Representative immunohistochemistry staining images of VEGFA at the marginal region of wounds in the Cut, SDDS and TDS groups at different time points. **(f)** Statistical analysis of the expression of VEGFA at the marginal region of wounds in the Cut, SDDS and TDS groups at different time points. *Cut *vs* SDDS, # Cut *vs* TDS, & SDDS *vs* TDS. **(g)** Representative immunohistochemistry staining images of VEGFA at the marginal region of wounds in the Cut, SDDS and TDS groups at different time points. Scale bar: 100μm and 50μm, magnification times: 40× and 100×. **(h)** Statistical analysis of the expression of VEGFA at the middle region of wounds in the Cut, SDDS and TDS groups at different time points. *Cut *vs* SDDS, #Cut *vs* TDS, &SDDS *vs* TDS. All data from three independent experiments were presented as mean ± SD. ^*^p < 0.05, ^**^p < 0.01, ^***^p < 0.001, ^****^p < 0.0001; ^#^*p* < 0.05, ^##^*p* < 0.01, ^###^*p* < 0.001, ^####^*p* < 0.0001; ^&^*p* < 0.05, ^&&^*p* < 0.01. *SDDS* second-degree deep scald, *TDS* third-degree scald, *H&E* hematoxylin and eosin, *VEGFA* vascular endothelial growth factor A

In order to determine the reason for impaired wound repair in the SDDS and TDS groups, the neovascularization was identified among the three groups. The expression of CD31 in granulation tissue, a marker of angiogenesis, was evaluated by immunohistochemistry staining. This was detected at the marginal and middle region of wounds at days 3, 7, 15 and 27 post-surgery, respectively (Figures 2a, c). These present data demonstrated that the number of blood vessels (the positive area of CD31) in the marginal area of wounds was significantly greater in the Cut group, when compared with the SDDS and TDS groups, at day 7 post-surgery, and this was followed by a remarkable decline. However, it was observed that the number of blood vessels in the SDDS and TDS groups surpassed that in the Cut group at day 15 (Figure 2b). In the middle region of wounds, the quantity of blood vessels for both SDDS and TDS groups reached a peak at day 15, and this was significantly lower than that of the Cut group at days 15 and 27 following damage (Figure 2d). This implies an impairment of vascularization in both SDDS and TDS groups. Furthermore, VEGFA, which is another marker of angiogenesis, was also tested by immunohistochemistry staining at the marginal and middle region of the wounds at days 3, 7, 15 and 27 after injury (Figures 2e, g). According to the number of blood vessels, the expression of VEGFA in the marginal region of wounds was significantly greater in the Cut group, when compared with the SDDS and TDS groups, and this peaked at day 7 after injury, while that in the SDDS and TDS groups sequentially reached a maximum at day 15 (Figure 2f). As shown in Figure 2h, it was observed that the expression of VEGFA in the middle region of wounds was significantly higher in the Cut group, when compared with the SDDS and TDS groups at day 3, 7, 15 and 27, and this reached a peak at day 15 after damage. These results revealed that obstruction of the formation of blood vessels occurred in the SDDS and TDS groups, when compared with the Cut group.

### The SDDS group was characterized by better healing quality when compared with the Cut and TDS groups

The immunohistochemistry of α-SMA was administered to analyze the number of myofibroblasts at days 3, 7, 15 and 27 after cutaneous damage (Figures 3a, e). The present data revealed that the number of myofibroblasts located at the marginal region of wounds was significantly greater in the Cut group, when compared with the SDDS and TDS groups, at day 7 after injury, and this reached a maximum at day 15 (Figure 3b). Furthermore, the present results revealed that the number of myofibroblasts in the middle region of wounds was significantly greater in the Cut group, when compared with the SDDS and TDS groups, and this peaked at day 15 post-surgery (Figure 3d). Then, the expression of TGF-β in the Cut, SDDS and TDS groups was assayed by immunohistochemistry at days 3, 7, 15 and 27 (Figures 3e, g). As shown in Figure 3f, the expression of TGF-β at the marginal region of wounds was significantly higher at days 3 and 7 in the Cut group, and this reached a peak at day 7, when compared with the SDDS and TDS groups, while this was markedly higher in the SDDS and TDS groups, when compared with the Cut group, and reached a maximum at day 15 after damage. The present data also demonstrate that the expression of TGF-β in the middle region of wounds was significantly higher in the Cut and SDDS groups, when compared with the TDS group, and this peaked at day 15 after damage (Figure 3h). The aforementioned results indicate that the proliferation of myofibroblasts was impaired in the SDDS and TDS groups, when compared with the Cut group.

**Figure 3.**
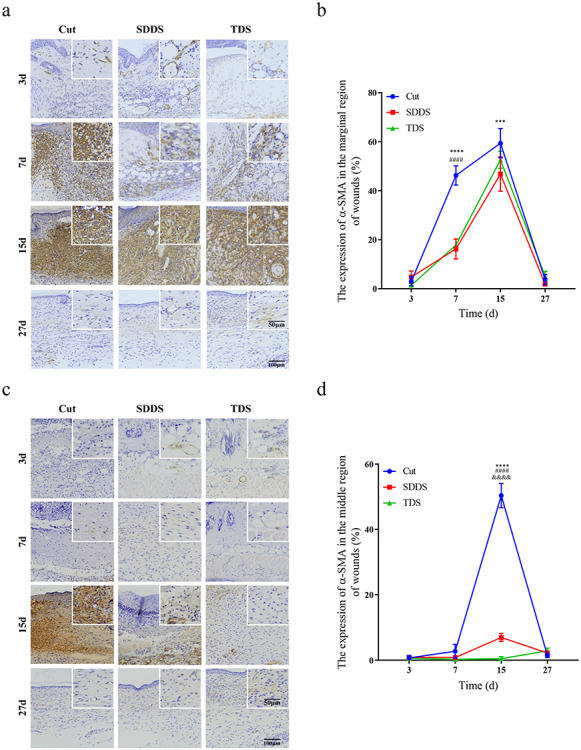
The wound-healing quality in the Cut, SDDS and TDS groups at day 15 after cutaneous damage. **(a)** Representative immunohistochemistry staining images of α-SMA in the marginal region of wounds in the Cut, SDDS and TDS groups. Scale bar: 50 and 100μm, magnification times: 40× and 100×. **(b)** Statistical analysis of the expression of α-SMA in the marginal region of wounds at day 15. *Cut *vs* SDDS, #Cut *vs* TDS, &SDDS *vs* TDS. **(c)** Representative immunohistochemistry staining images of α-SMA in the middle region of wounds in the Cut, SDDS and TDS groups. Scale bar: 50 and 100μm, magnification times: 40× and 100×. **(d)** Statistical analysis of the expression of α-SMA in the middle region of wounds at day 15. *Cut *vs* SDDS, #Cut *vs* TDS, & SDDS *vs* TDS. **(e)** Representative immunohistochemistry staining images of TGF-β in the marginal region of wounds in the Cut, SDDS and TDS groups. **(f)** Statistical analysis of the expression of VEGFA in the marginal region of wounds at day 15. *Cut *vs* SDDS, #Cut *vs* TDS, &SDDS *vs* TDS. **(g)** Representative immunohistochemistry staining images of TGF-β in the middle region of wounds in the Cut, SDDS and TDS groups. Scale bar: 50 and 100μm, magnification times: 40× and 100×. **(h)** Statistical analysis of the expression of VEGFA in the middle region of wounds at day 15. #Cut *vs* TDS, &SDDS *vs* TDS. **(i)** Representative images of Masson and Sirius red staining on the 15th day among the three groups. Scale bar: 500 and 20μm, magnification times: 10× and 100×. **(j)** Statistical analysis of the collagen content; **(k)** the ratio of collagen I/III; and **(l)** the percentage of collagen in the Cut, SDDS and TDS groups. **(m)** Representative images of Gordon-Sweets and Victoria blue staining in the Cut, SDDS and TDS groups. Scale bar: 500 and 20μm, magnification times: 10× and 100×

**Figure 3.**
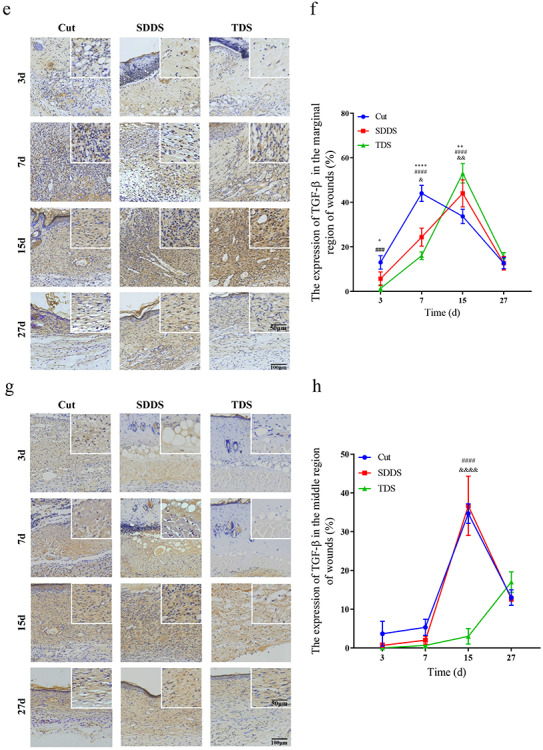

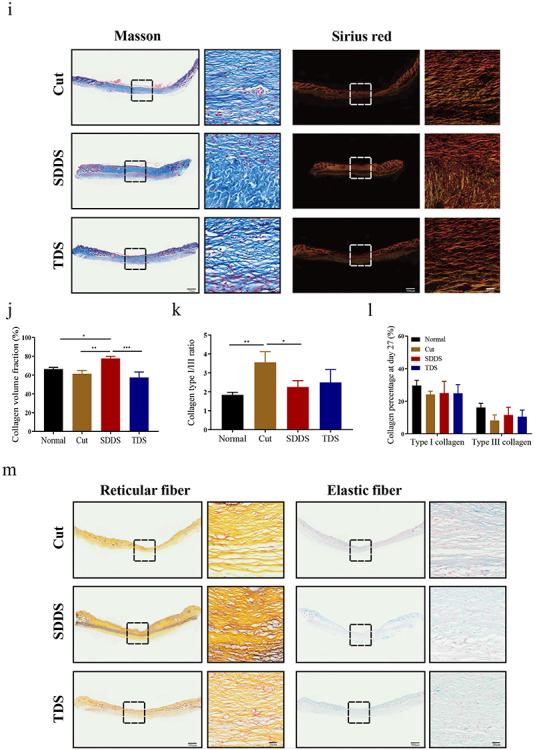

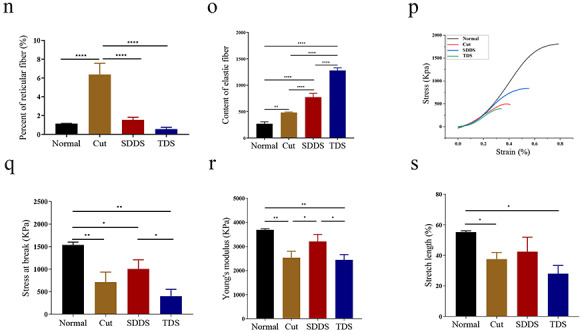
(Continued) **(n)** Statistical analysis of the area of the reticular fiber; and **(o)** the elastic fiber in the normal, Cut, SDDS and TDS groups. Scale bar: 500 and 20 μm. **(p)** Representative images of tensile failure curve, failure stress, Young’s modulus and failure length in the normal, Cut, SDDS and TDS groups. **(q)** Statistical analysis of stress at break, **(r)** Young’s modulus and **(s)** stress length in the normal, Cut, SDDS and TDS groups. *Cut *vs* SDDS. All data from three independent experiments were presented as mean ± SD. ^*^p < 0.05, ^**^p < 0.01, ^***^p < 0.001, ^****^p < 0.0001; ^#^*p* < 0.05, ^##^*p* < 0.01, ^###^*p* < 0.001, ^####^*p* < 0.0001; ^*^p < 0.05, ^**^p < 0.01, ^****^p < 0.0001. *SDDS* second-degree deep scald, *TDS* third-degree scald, *VEGFA* vascular endothelial growth factor A, α-*SMA* α-smooth muscle actin, *TGF*-β transformation growth factor-β

In order to determine the healing quality among the three groups, the normal mice, seen as the normal group, were utilized as the standard indictor. Next, the investigators further analyzed the expression of collagen in the normal (data not shown), Cut, SDDS and TDS groups at day 27 after skin injury (Figure 3i). The Masson staining results revealed that the expression of collagen was significantly higher in the SDDS group, when compared with the Cut and TDS groups; however, the expression of collagen in the Cut group was more similar to the normal group, when compared with the SDDS and TDS groups (Figure 3j). The Sirius red staining results demonstrated that the content of type I collagen exceeded that of type III collagen among the three groups (Figure 3l), and the percent of type I to III collagen was higher in the Cut group, when compared with the SDDS and TDS groups, while the ratio of type I to III collagen in the SDDS group was closer to the normal group, when compared with the Cut and TDS groups (Figure 3k). In addition, the expression of the reticular fibers and elastic fibers was assayed in the normal (data not shown), Cut, SDDS and TDS groups by Gordon-Sweets staining and Victoria blue staining, respectively (Figure 3m). The Gordon-Sweets staining results revealed that the percent of reticular fibers was significantly higher in the Cut group, when compared with the SDDS and TDS groups (Figure 3n). Furthermore, the data revealed that the content of elastic fibers was greater in the TDS group, when compared with the Cut and SDDS groups (Figure 3o).

It has been considered that the biomechanical behavior of the skin largely reflects the quality of healing during wound repair. Hence, the investigators assessed the mechanical properties of the skin during wound healing via tensile failure (Figure 3p). The results demonstrated that stress at break (Figure 3q), Young’s modulus (Figure 3r) and stretch length (Figure 3s) were closer to the normal group, when compared with the Cut and TDS groups. Taken together, these results suggested that the quality of healing was better in the SDDS group, when compared with the Cut and TDS groups.

### Impairment of inflammatory response in the SDDS and TDS groups when compared with the Cut group

In order to determine when the inflammatory response was involved in wound healing after cutaneous injury, H&E staining was performed to determine the number of inflammatory cells after cutaneous injury at days 3, 7, 15 and 27, respectively (Figures 4a, c). The present results demonstrated that the amount of inflammation cells in the marginal region of wounds was greater in the Cut group, when compared with the SDDS and TDS groups, after injury, and this reached a peak at day 7, while it reached the maximum at day 15 in the SDDS and TDS groups (Figure 4b), indicating that the infiltration of inflammatory cells was delayed in the SDDS and TDS groups, when compared with the Cut group. In the middle region of wounds, the present data revealed that the number of inflammation cells was greater in the Cut group, when compared with the SDDS and TDS groups, after surgery, and this reached a peak at day 15, followed by a decrease (Figure 4d). To verify the results, immunohistochemistry staining for CD45 was used to analyze the inflammatory response in the Cut, SDDS and TDS groups **(**Figures S1a, c). The data showed that the expression of CD45 in the marginal region of wounds in the Cut group was higher, and reached a maximum at day 7, when compared with the other two groups, while it reached a peak in the SDDS and TDS groups at day 15, when compared with the Cut group **(**Figure S1b). Furthermore, the present data revealed that, in the middle region of wounds, the expression of CD45 in the Cut group was greater, when compared with the SDDS and TDS groups, followed by a reduction, after injury **(**Figure S1d**)**. Next, the expression of IFN-γ at days 3, 7, 15 and 27 was detected by immunohistochemistry staining after injury (Figures 4e, g). The present data demonstrate that the expression of IFN-γ in the marginal region of wounds reached a peak at day 15 in the Cut, SDDS and TDS groups, and this was significantly higher in the Cut group, when compared with the other two groups (Figure 4f). Moreover, immunohistochemistry results also revealed that the expression of IFN-γ in the middle region of wounds was markedly higher at days 7 and 15 following injury in the Cut group, when compared with the SDDS and TDS groups, and this reached a maximum at day 15, followed by a decrease (Figure 4h). Next, the expression of IL-17 was analyzed by immunohistochemistry at days 3, 7, 15 and 27, respectively (Figures 4i, k). It was proven that the expression of IL-17 in the marginal region of wounds reached a peak at day 7 after injury in the Cut and SDDS groups, and this was significantly higher in the Cut group, when compared with the TDS and SDDS groups (Figure 4j). As shown in Figure 4l, the results demonstrated that the expression of IL-17 in the middle region of wounds was significantly higher at post-surgery in the Cut group, when compared with the SDDS and TDS groups, and this reached a peak at day 15. These results suggest that compared with the Cut group, the inflammatory response was postponed in the SDDS and TDS groups after surgery.

**Figure 4.**
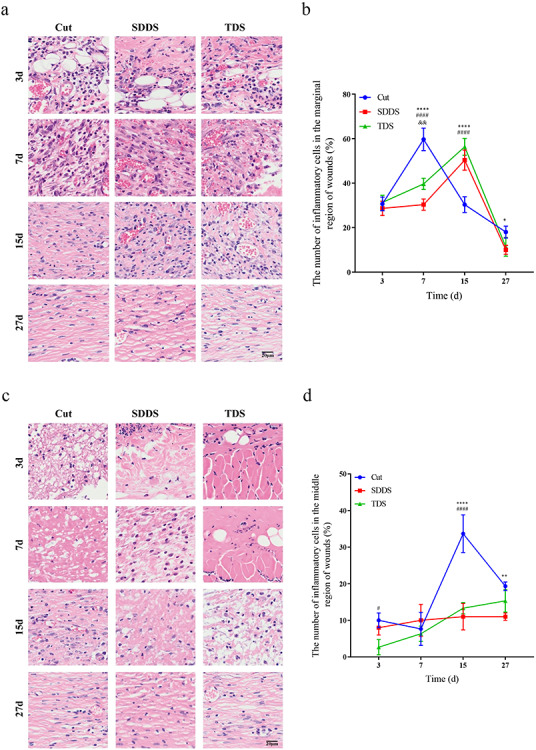
The inflammatory response at days 3, 7, 15 and 27 in the Cut, SDDS and TDS groups after procedure. **(a)** The distribution of inflammatory cells in the marginal region of wounds at different time points in the Cut, SDDS and TDS groups. Scale bar: 20μm, magnification times: 100×. **(b)** Statistical analysis of the quantity of inflammatory cells in the marginal region of wounds in the Cut, SDDS and TDS groups during skin healing. *Cut *vs* SDDS, #Cut *vs* TDS, &SDDS *vs* TDS. **(c)** H&E staining showed that the distribution of inflammatory cells at the middle region of wounds at different time points in the Cut, SDDS and TDS groups. Scale bar: 20μm, magnification times: 100×. **(d)** Statistical analysis of the quantity of inflammatory cells at the middle region of wounds in the Cut, SDDS and TDS groups during skin healing. *Cut *vs* SDDS, # Cut *vs* TDS. **(e)** Representative images of IFN-γ by immunohistochemistry staining in the marginal region of wounds at different days in the Cut, SDDS and TDS groups. Scale bar: 100μm and 50μm, magnification times: 40× and 100×. **(f)** Statistical analysis of the expression of IFN-γ in the marginal region of wounds in the Cut, and SDDS and TDS groups. *Cut *vs* SDDS, #Cut *vs* TDS, &SDDS *vs* TDS. **(g)** Immunohistochemistry staining showed that the expression of IFN-γ in the middle region of wounds at different days in the Cut, SDDS and TDS groups. Scale bar: 100μm and 50μm, magnification times: 40× and 100×. **(h)** Statistical analysis of the expression of IFN-γ in the middle region of wounds in the Cut, and SDDS and TDS groups. *Cut *vs* SDDS, #Cut *vs* TDS. **(i)** Representative immunohistochemistry staining pictures of IL-17 in the marginal region of wounds among the three groups during wound healing. Scale bar: 100μm and 50μm, magnification times: 40× and 100×

**Figure 4.**
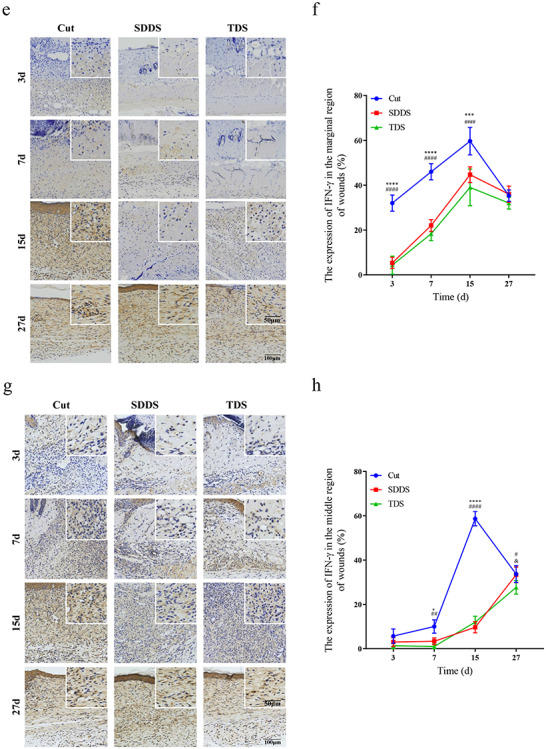

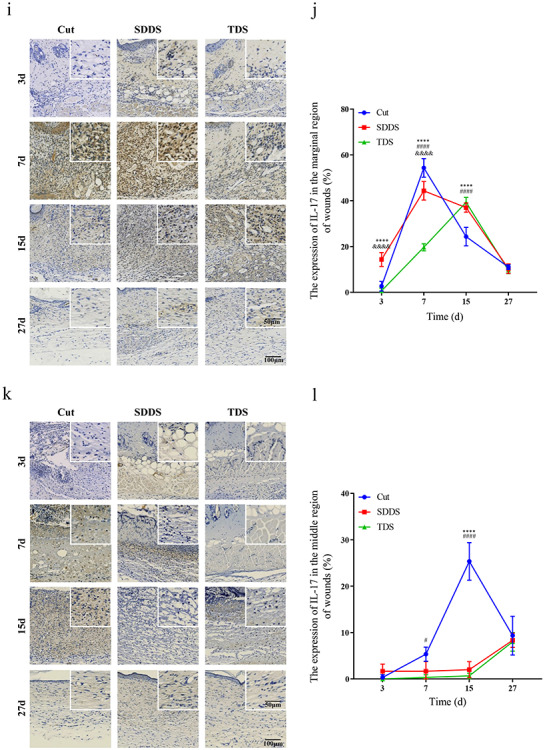
(Continued) **(j)** Statistical analysis of the expression of IL-17 in the marginal region of wounds in the Cut, SDDS and TDS groups. *Cut *vs* SDDS, #Cut *vs* TDS, &SDDS *vs* TDS. **(k)** Representative images of IL-17 by immunohistochemistry staining at the middle region of wounds among three groups during wound healing. Scale bar: 100μm and 50μm, magnification times: 40× and 100×. **(l)** Statistical analysis of the expression of IL-17 at the middle region of wounds in the Cut, SDDS and TDS groups. *Cut *vs* SDDS, #Cut *vs* TDS. All data from three independent experiments were presented as mean ± SD. ^*^p < 0.05, ^**^p < 0.01, ^***^p < 0.001, ^****^p < 0.0001; ^#^*p* < 0.05, ^##^*p* < 0.01, ^###^*p* < 0.001, ^####^*p* < 0.0001; ^&^*p* < 0.05, ^&&^*p* < 0.01, ^&&&&^*p* < 0.0001. *SDDS* second-degree deep scald, *TDS* third-degree scald, *IL-17A* interleukin 17A, *IFN-γ* interferon γ

## Discussion

Delayed wound healing following accidental trauma continues to have serious, essential, unresolved obstacles for clinicians and researchers. In general, the healing of cutaneous wounds is characterized by multiple overlapping and successive processes, such as hemostasis, inflammation, proliferation and remodeling^[^^4^^]^. Patients who have undergone burn scald damage are usually confronted with the impairment of skin wound healing, which may result in continuous ulceration, as observed in diabetic patients^[^^14^^]^. However, to date, the detailed mechanism of wound healing in burn scald injury has remained vague. Consequently, the investigators performed a systematic assessment of both SDDS and TDS in the present study, which was carried out by assaying indicators, including the wound-healing rate, re-epithelialization, the formation of granulation tissue, vascularization, the mechanical properties of the wound, collagen synthesis, and inflammatory infiltration. It was initially demonstrated that healing of the cutaneous wound was significantly postponed in the SDDS and TDS groups, when compared with the Cut group.

Re-epithelialization and the formation of granulation tissue are believed to be required for efficient wound closure after skin injury^[^^15^^]^. Granulation tissue mainly comprises three kinds of cells: endothelial cells, fibroblasts, and a number of inflammatory cells, such as macrophages and lymphocytes. These facilitate the formation of re-epithelialization^[^^16^^]^. The process of re-epithelization follows the filling of granulation tissue to the center and marginal region of the wound, which is characterized by the proliferation and migration of keratinocytes toward the core part of the lesion^[^^17^^]^. As a consequence, granulation tissue directly affects the speed and quality of re-epithelialization, and further influences the healing speed of cutaneous wounds. A previous study conducted by the investigators revealed that platelet-rich plasma accelerated skin wound healing by promoting angiogenesis, the formation of granulation tissue and re-epithelialization in a mouse model^[^^18^^]^. In the present study, it was observed that the formation of granulation tissue was impaired in the SDDS and TDS groups, when compared with the Cut group, which may lead to poor re-epithelialization and wound-healing rate.

Angiogenesis is the key element for promoting wound healing, and this is commonly used to evaluate the quality of wound repair^[^^19^^]^. The endothelium, which is comprised of endothelial cells, acts as a barrier between the blood and other components of the blood vessels, and is one of the most important contributors to angiogenesis^[^^20^^]^. Endothelial cells are involved in the formation of new blood vessels, which help deliver oxygen and nutrients to the wound site, allowing keratinocytes and fibroblasts to grow, proliferate, migrate, generate new epidermis, or synthesize collagen, ultimately leading to wound healing^[^^21^^]^. It has been commonly considered that the number of blood vessels in cutaneous wounds is one of the most important factors for the formation of granulation tissue, further accelerating wound repair^[^^22^^]^. Several studies have shown that enhancing neovascularization rapidly promotes wound repair and dramatically improves the quality of the wound, suggesting that vascularization is closely correlated to wound healing^[^^23^^]^. For example, in the mouse with diabetic wound model, exosomes from human urine-derived stem cells (USC-Exos) were used to investigate wound healing, and it was discovered that USC-Exos could accelerate wound healing and augment the functional properties of wound healing-related cells including the angiogenic activities of endothelial cells. Further functional assays have shown that deletion in the malignant brain tumor 1 (DMBT1) protein is required for USC-Exo-induced promotion of angiogenic response of cultured endothelial cells, as well as angiogenesis and wound healing in diabetic mice^[^^24^^]^. Similarly, in another study, Whittam et al.^[^^25^^]^ reported that the dipeptidyl peptidase-4 (DPP-4) inhibitor, MK0626, significantly accelerated wound healing, and increased wound vascularity, stromal cell-derived factor-1 (SDF-1) expression, and dermal thickness in diabetic wounds. In addition, the MK0626 treatment increased the number of bone marrow-derived mesenchymal progenitor cells (BM-MPCs) present in the bone marrow and in diabetic wounds. However, this had no effect on the BM-MPC population dynamics. BM-MPCs harvested from MK0626-treated mice exhibited increased chemotaxis in response to SDF-1, when compared with diabetic controls. The treatment with a DPP-4 inhibitor significantly improved wound healing, angiogenesis and endogenous progenitor cell recruitment in the setting of diabetes. Furthermore, the data revealed that postponed angiogenesis occurred in the SDDS and TDS groups, when compared with the Cut group, which may have been the cause of the impairment of granulation tissue formation.

In addition to angiogenesis, the wound contraction that mainly results from fibroblasts is also an important phase during wound repair after skin damage^[^^26^^,^^28^^]^. It has been accepted that efficient wound contraction is greatly beneficial for accelerating wound healing. However, excessive contraction is seriously harmful for injured tissue remolding, and further contributes to the formation of scars^[^^29^^,^^30^^]^. Accordingly, it was speculated that these mechanical properties may change in parallel with wound contraction. Interestingly, as expected by the investigators, the present data revealed that the mechanical properties of the SDDS group were greater, when compared with the other two groups, suggesting that the wound-healing quality in the SDDS group was better compared with the Cut and TDS groups. Due to the difference in mechanical properties, the shrinkage ability of fibrocytes may vary, which further affects the healing quality of wounds. Therefore, it may be promising in the future to evaluate the quality of wound repair from the perspective of mechanical properties. The possible mechanisms of fiber synthesis and contraction need to be explored further and elucidated.

In addition to wound contraction, collagen deposition is also an important process to determine the quality of wound healing, which partly affects the formation of scars^[^^31^^]^. During the complex process of wound closure, several pro-regenerative physiological interactions are mediated by collagen^[^^32^^]^. Collagen type I and III are the most abundant components of the extracellular matrix (ECM) of the dermal layer of the skin^[^^33^^]^. Specifically, collagen type I constitutes 80–85% of the dermal ECM, while collagen III constitutes 8–11%^[^^15^^]^. In the present research, the ratio of collagen I to collagen III was used to evaluate the quality of wound healing. To our knowledge, moderated collagen coordinates the interaction of growth factors, cytokines and chemokines, which affects cell behavior as a consequence of their binding to specific cell surface receptors or ECM proteins^[^^34^^,^^35^^]^. Collagen is also capable of interaction with a mass of regenerative pathways utilized in the process of skin wound healing, ranging from angiogenesis to re-epithelialization, but excessive collagen influences the quality of wound healing^[^^36^^]^. In the present study, the investigators utilized models of Cut, SDDS and TDS in mice to assess the collagen changes, and the ratio of collagen I to collagen III in the SDDS group was revealed to be closer to that of the normal group, but the underlying mechanism remained vague.

Numerous studies have uncovered that wound repair is a series of dynamic and complex physiological responses, which are simultaneously accompanied by inflammation and anti-inflammation factors, such as IL-1α, IL-1β, IL-17A, IFN-γ and TGF-β in addition to growth factors, hormones and other types of factors^[^^37–40^^]^. It has been demonstrated that in the human body, there is a relative balance between inflammatory response and anti-inflammatory response under normal conditions^[^^41^^]^. Nevertheless, when the skin is seriously damaged by an accident, continuous inflammatory infiltration occurs throughout the period of wound healing. Although a slight inflammatory response is helpful for promoting wound healing, persistent and immoderate inflammatory infiltration is harmful for the body due to the unbalance between inflammatory response and anti-inflammatory response^[^^42^^]^. Embryonic stem cell extracts (EXTs) were employed to investigate the role of the progression of wound healing in diabetic mice, and it was revealed that the *in vivo* topical administration of EXTs facilitates wound closure, contraction and re-epithelialization. Furthermore, the EXTs reduced the number of wound-infiltrating CD45^+^ inflammatory cells and increased the rate of repair and angiogenesis, when compared with controls^[43]^. In the present study, it was found that the expression of inflammatory factors and inflammatory cell infiltration was deferred in the SDDS and TDS groups, when compared with the Cut group. This may be the reason for the impairment of subsequent biological events, including angiogenesis, the proliferation and migration of fibroblasts, the formation of granulation tissue, re-epithelization, and others.

Taken together, our data demonstrated that the SDDS group possessed the better healing quality, when compared with the Cut and TDS groups, which may be helpful in the prognosis for burn patients. Although there are still the possible molecular mechanisms of scald burn to be explored in more depth, the present study provides some guidance on the treatment of wound healing.

## Conclusions

In conclusion, the present study first administered a comprehensive analysis of both SDDS and TDS in *in vivo* experiments, which further proved the difference in the speed and quality of the three important stages of wound healing. Although there are still the underlying molecular mechanisms of both SDDS and TDS to be explored in more depth, our study provides some guidance for the treatment of wound healing.

## Data availability

The data and material to support the findings can be found in the Cut, SDDS and TDS groups through *in vivo* experiments, which further proved that the obstacle of the formation of granulation tissues leaded to the delayed wound healing after scald burn injury in mice.

## Supplementary Material

Supplementary_legend_tkab004

## Abbreviations

ANOVA: Analysis of variance; AOD: Average optical density; BM-MPCs: Bone marrow-derived mesenchymal progenitor cells; DMBT1: Deletion in the malignant brain tumor 1; DPP-4: Dipeptidyl peptidase-4; ECM: Extracellular matrix; EXTs: Embryonic stem cell extracts; IL-1a: Interleukin 1a; IL-1β: Interleukin 1β; IL-17A: Interleukin 17A; IFN-γ: Interferon γ SDDS: Second-degree deep scald; SDF-1: Stromal cell-derived factor-1; SMA: Smooth muscle actin; TDS: Third-degree scald; TGF: Transforming growth factor; USC-Exos: Exosomes from human Urine-derived stem cells; VEGFA: Vascular endothelial growth factor A.
