# Supplementary material for: Obstruction of the formation of granulation tissue leads to delayed wound healing after scald burn injury in mice
Source: Burns Trauma. 2021 Apr 29;9:tkab004. doi: 10.1093/burnst/tkab004 (PMC8240558; doi:10.1093/burnst/tkab004)
Supplement: Supplementary_legend_tkab004 [file supplementary_legend_tkab004.docx]

***Supplementary legend:***

**Fig S4.** **The inflammatory response at days 3, 7, 15 and 27 in the cut, SDDS and TDS groups after procedure.** (a) The expression of CD45 at the wound margin and (c) in the middle wound at different time points in the cut, SDDS and TDS groups. (b) Statistical analysis of the expression of CD45 of wound margin and (d) the middle wound in the cut, SDDS and TDS groups during skin healing. Scale bar: 100µm. ^*^ means Cut *vs* SDDS, ^#^ means Cut *vs* TDS, ^&^ means SDDS *vs* TDS. All data from three independent experiments were presented as mean±SD. **p ＜0.01, ****p ＜0.0001; ^####^p ＜0.0001; ^&&^p ＜0.01.
